# Isoform level expression profiles provide better cancer signatures than gene level expression profiles

**DOI:** 10.1186/gm437

**Published:** 2013-04-17

**Authors:** ZhongFa Zhang, Sharmistha Pal, Yingtao Bi, Julia Tchou, Ramana V Davuluri

**Affiliations:** 1 Center for Systems and Computational Biology, Molecular and Cellular Oncogenesis Program, The Wistar Institute, Philadelphia, PA 19104, USA; 2 Department of Surgery, Abramson Cancer Center, University of Pennsylvania School of Medicine, Philadelphia, PA 19104, USA

## Abstract

**Background:**

The majority of mammalian genes generate multiple transcript variants and protein isoforms through alternative transcription and/or alternative splicing, and the dynamic changes at the transcript/isoform level between non-oncogenic and cancer cells remain largely unexplored. We hypothesized that isoform level expression profiles would be better than gene level expression profiles at discriminating between non-oncogenic and cancer cellsgene level.

**Methods:**

We analyzed 160 Affymetrix exon-array datasets, comprising cell lines of non-oncogenic or oncogenic tissue origins. We obtained the transcript-level and gene level expression estimates, and used unsupervised and supervised clustering algorithms to study the profile similarity between the samples at both gene and isoform levels.

**Results:**

Hierarchical clustering, based on isoform level expressions, effectively grouped the non-oncogenic and oncogenic cell lines with a virtually perfect homogeneity-grouping rate (97.5%), regardless of the tissue origin of the cell lines. However, gene levelthis rate was much lower, being 75% at best based on the gene level expressions. Statistical analyses of the difference between cancer and non-oncogenic samples identified the existence of numerous genes with differentially expressed isoforms, which otherwise were not significant at the gene level. We also found that canonical pathways of protein ubiquitination, purine metabolism, and breast-cancer regulation by stathmin1 were significantly enriched among genes thatshow differential expression at isoform level but not at gene level.

**Conclusions:**

In summary, cancer cell lines, regardless of their tissue of origin, can be effectively discriminated from non-cancer cell lines at isoform level, but not at gene level. This study suggests the existence of an isoform signature, rather than a gene signature, which could be used to distinguish cancer cells from normal cells.

## Background

The past decade has witnessed unprecedented developments in high-throughput technologies, and their application has led to the molecular classification of many cancers [1]. Molecular profiling of gene expression, using microarrays, has shown that heterogeneity in outcome and survival of patients with cancer can be explained, in part, by genomic variation within the primary tumor. These technologies have helped identify many genetic and epigenetic modifications involved in the initiation and progression of various cancers, but their precise molecular mechanisms remain unclear. Furthermore, novel drugs have been developed to target some of the molecular pathways underlying the carcinogenic processes and maintenance of cancer phenotypes [2,3] yet, these drugs have provided limited survival benefits to only a small subset of patients with cancer, and only a small number of practically useful biomarkers are presently available. Improved molecular classification of cancers is essential to identify highly sensitive and specific biomarkers and therapeutic targets that reflect the molecular mechanisms functionally involved in tumor type-specific survival, drug resistance, tumor relapse, and patient outcome [4].

One of the reasons for the limited success in the quest for genomic-based, personalized medicine is the assumption of a 'one gene → one protein → one functional pathway' paradigm in most of the studies investigating molecular classification or therapeutic targets for cancer [5]. Recently, by making use of chromatin immunoprecipitation sequencing (ChIP-seq) and mRNA sequencing (mRNA-seq) approaches, we and others have discovered widespread use of alternative promoters and alternative splicing in mammalian genes in various tissues, developmental stages, and cell lines [6-9]. In fact, numerous genes displaying complex gene regulation via use of alternative promoters and alternative splicing, have been known for some time, and recent evidence suggests that almost all multi-exon human genes have more than one mRNA isoform. During alternative splicing, the coding and non-coding regions of a single gene are rearranged to generate several messenger RNA transcripts, yielding distinct protein isoforms with differing biological functions. Notably, there is growing evidence linking aberrant use of alternative mRNA isoforms with cancer formation; several oncogenes and tumor-suppressor genes (for example, *LEF1, TP63, TP73, HNF4A, RASSF1*, and *BCL2L1*) are already known to have multiple promoters and alternative splice forms [10-16]. Moreover, it is known that the aberrant use of one isoform over another in some of these genes is directly linked to cancer cell growth [17]. Although the prevalence of alternative splicing in cancer genomes has been discussed in the literature [18-20], and it has been shown that use of splice forms provides better classification of normal and cancerous prostate tissue, it is not clear whether the use of genome-wide isoform level gene-expression profiles can provide a better global discriminative signature for cancer and normal cells.

Microarray expression profiling remains a powerful tool for identifying different subtypes of cancers. However, almost all microarray-based studies reported to date have measured the expression of the gene at gene level in a given locus, although a few exceptions in recent years have used exon arrays to measure differences at the exon and/or at transcript variant level. The recent application of exon arrays [21] and the advent of massive parallel sequencing is allowing whole cancer genomes and transcriptomes to be sequenced with extraordinary speed and accuracy, providing insight into the bewildering complexity of isoform level expression of gene transcripts [7]. The Encyclopedia of DNA Elements (ENCODE) consortium, a collective effort to facilitate and accelerate the annotation of functional elements in the human genome, is generating genome-wide expression data in various human cell lines through the use of exon microarrays [20]. Among the available data are gene-expression datasets, generated by the ENCODE consortium using an Affymetrix platform (GeneChip Human Exon 1.0 ST Array), across various cell lines that can be classified as either oncogenic (tumor/cancer) or non-oncogenic (normal). The arrays interrogate transcripts across their entire length, which can detect splicing differences between various types of samples [22-24]. Exons within a gene are represented on the microarray by multiple probe sets. The exon expression can thus be obtained by summarizing all the probe sets for this exon on the microarray. Once the exon-level expressions are obtained, the individual transcript expression of the gene and the total expression of the gene itself then can be inferred from the calculated exon expression, based on assumptions that the isoform structures and number of isoforms of the gene are known beforehand.

With genome-wide isoform level and gene level expression profiles in hand, it is natural to ask how the isoform level expression profiles of different oncogenic and non-oncogenic samples will cluster together, and whether isoform level expression profiles can lead to more accurate discriminators between oncogenic and non-oncogenic samples compared with gene level expression profiles. If the answer is yes, it is important to know which genes and pathways contribute to the improvement of discrimination at isoform level compared with gene level.

In the present study we analyzed Affymetrix exon-array data-sets collected from the public domain, primarily the ENCODE project from the National Center for Biotechnology Information (NCBI) Gene Expression Omnibus (GEO) database, which comprises 160 datasets from various cell lines of either non-oncogenic or oncogenic tissue origin. These data-sets were used to test the hypothesis that isoform level expression analysis provides abetter discriminator between non-oncogenic and oncogenic cell types than gene level expression analysis.

## Methods

### Summary of exon-array datasets

Unprocessed gene-expression datasets, generated using a whole-transcript GeneChip platform (Human Exon 1.0 ST Array; Affymetrix Inc., Santa Clara, CA, USA), were downloaded from the GEO public data depository, deposited mainly by the ENCODE project[18]. The GEO records GSE15805 [25], GSE17778, GSE19090 [26] and GSE17349 [27] contain, respectively, 79, 36, 83, and 8 samples of various cell lines. After excluding samples that were related to blood, progeria fibroblast, and stem cells, we had a total of 160 exon-array datasets, corresponding to 87 non-oncogenic and 73 oncogenic cell lines of various tissue origins. From the 160 datasets included in the analysis, we used 8 melanoma samples and 4 non-oncogenic melanocyte samples to form the first matched non-oncogenic and oncogenic pair, and used 4 datasets representing non-oncogenic human mammary epithelial cells (HMEC) and 8 datasets from a human breast adenocarcinoma cell line (MCF7) to form the second matched non-oncogenic and oncogenic pair. The complete classification and labeling information of cell lines used in this study are summarized in the supplementary information (see Additional file 1: Table S1).

### Estimation of isoform level and gene level expression values from exon-array data

The isoform level (transcript-level) and gene level expression estimates were obtained by the Multi-Mapping Bayesian Gene eXpression (MMBGX) algorithm for Affymetrix whole-transcript arrays [28], based on the Ensembl database (version 56) [29], which contains a total of 114,930 different transcript annotations that correspond to 35,612 different gene models. We set the burn-in iteration at 8,192 and real iteration at 16,384 for both gene and isoform levels. All other parameters were set to their default values in the stand-alone algorithm. The algorithm gave a stable estimation of both gene level and isoform level expressions. For example, two independent runs on the same sample provided almost identical expression levels even with different seeds for the algorithm (correlation coefficient > 0.999, data not shown), whereas runs on different samples gave comparable results, but with much lower correlation (correlation coefficient of about 0.97). Expression estimates across all the samples were then normalized using the locally weighted scatterplot smoothing (*loess*) algorithm [30,31], also incorporated in the package.

### Clustering and pathway analyses

We used the general hierarchical cluster algorithm to cluster the samples, using Euclidean distance as a measurement for dissimilarities [32]. We also applied consensus hierarchical clustering to assess the stability of the clustering results by multiple runs of the clustering algorithm on resampled data [32,33], and calculated consensus index as reported previously [33]. Briefly, the consensus index is defined for each pair of samples, that is, the consensus index of sample pair (i, j) records the number of times that samples i and j are assigned to the same cluster, divided by the total number of times both samples are selected. To find the differential genes between two conditions, we used the *limma *method [34,35]. An isoform or gene was selected if both its fold change was greater than a cut-off value of 2, and the false discovery rate (FDR)-adjusted *P *value was smaller than a cut-off value of 0.01 for all comparisons between the two conditions. Ingenuity Pathway Analysis (IPA) [36] was used to associate the identified gene sets with biological functions, canonical pathways, and networks. To identify pathway differences arising from gene sets identified at either isoform or gene level, we used the counting method on the *P *values of pathways from the IPA analysis; the *P *values were used as an indicator of association strength between the gene sets and pathways. For the three pairwise oncogenic/non-oncogenic comparisons (all oncogenic cell lines versus non-oncogenic cell lines, melanocyte versus melanoma, HMEC versus MCF7), a pathway was selected and reported if it was significantly associated with the gene sets identified at isoform-level in all three pairs of comparisons, but was not significantly associated with the gene sets identified at gene-level in all three pairs of comparisons, or *vice versa*. The significance level was set at *P *< 0.05 for all comparisons. All calculations were performed using Bioconductor (version 2.8 or above; Open Source software for bioinformatics, http://www.bioconductor.org) and R platform (version 2.10; The R Project for Statistical Computing, http://www.r-project.org) [37].

### Ethics approval

The study protocol was approved by the institutional review board, and all data collection and analyses adhered to the protocols approved by the institutional review board. Informed consent was obtained from all participants.

### Clinical characteristics of study cohort

Women with primary operable breast cancer undergoing breast surgery at the Hospital of the University of Pennsylvania were asked to participate in our tissue-banking protocol. The study cohort included four women diagnosed with breast cancer between 2010 and 2011. Clinical characteristics, including age at diagnosis, ethnicity, histology, tumor size, tumor grade, and number of involved (positive) axilla nodes are provided (see Additional file 2: Table S2A).

### Sample collection

After completion of surgery, the breast cancer within the surgical specimen was examined by surgical pathologists. Upon completion of gross examination and inking of the tumor specimen, fresh tumor tissue was taken from the center of the tumor without interfering with margin assessment as determined by the pathologists. A small portion of the tumor tissue and a small portion of normal adjacent breast tissue were collected, then immediately immersed in liquid nitrogen and stored at -80°C. RNA was isolated using this snap-frozen tumor tissue.

### RNA isolation and reverse transcriptase-quantitative PCR experiment

Expression of transcripts/isoforms for seven genes in HMEC, MCF7, MDA-MBA-231, and T47D cell lines and expression of two *TPM4 *isoforms in primary breast-cancer tissues were measured by reverse transcriptase -quantitativePCR (RT-qPCR). Total RNA from cells and tissues were using TRI reagent (Sigma-Aldrich Inc., St. Louis, MO, USA) in accordance with the manufacturer's instructions. For breast-cancer and normal breast tissues, up to 50 mg of frozen tissue was transferred to 1 ml of TRI reagent, then the tissue was immediately homogenized and RNA extraction protocol was followed. Briefly, 0.5 μg of total RNA was reverse-transcribed in a 20 μl reaction with SuperscriptII reverse transcriptase (Invitrogen Inc.) in accordance with the manufacturer's instructions. RT-qPCR was performed using a master mix (Power SYBR Green; Applied Biosystems Inc., Foster City, CA, USA) and fold expression was calculated using the 2^-ΔΔCT ^method. RT-qPCR results were normalized based on the expression of *GAPDH *for *TPM4 *and *TBP *for the other transcripts. The measured isoforms and the primers used for the isoform-specific PCR are presented (see Additional file 2: Table S2B).

## Results

### Clustering of samples using isoform level expression estimates provided more homogeneous grouping than gene level expression estimates of oncogenic and non-oncogenic cell lines gene level

**Figure 1:**
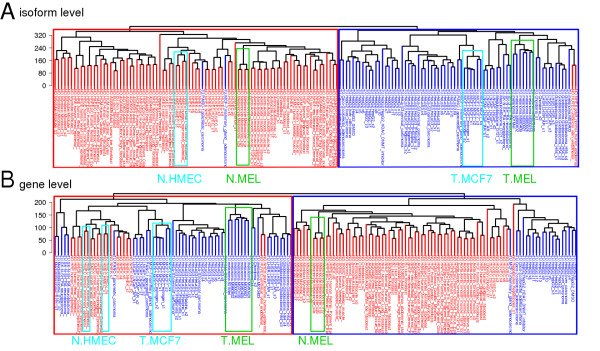
**Hierarchical clustering dendrograms of 160 datasets (73 oncogenic and 87 non-oncogenic) from cell lines of various tissue origins, using expression estimates of (A) 87,345 transcripts and (B) 27,063 genes**. The top 76% of genes/transcripts with the highest coefficient of variation (CV) of expression profile across all the samples was used for the clustering (see Additional file 3: Figures S1 and S2 for a series of dendrograms obtained by different CV cut-off points). The non-oncogenic melanocyte (N.Mel) and melanoma (T.Mel) cell lines were clustered together and separated into non-oncogenic and oncogenic groups in dendrogram A, whereas in dendrogram B, the N.HMEC samples were not grouped together and were clustered with the overall oncogenic group. N.HMEC is the normal breast cell line (human mammary epithelial cells) and T.MCF7 is the breast-cancer cell line MCF7.

Initial processing of the exon-array datasets generated expression estimates for a total of 114,930 different transcripts and 35,612 different genes in 160 different datasets or samples. To test our hypothesis that the isoform level expression profiles are better than the gene level expression profiles at discriminating non-oncogenic and cancer cellsgene level, we performed unsupervised clustering of 160 samples. Hierarchical clustering was performed by selecting the transcripts/genes showing the most variable expression, as determined by coefficient of variation for the estimated isoform-/gene level expression values across all samples. The dendrograms showed more homogeneous clustering of samples for isoform level expression analysis (Figure 1A) than for gene level expression analysis (Figure 1B). Similar clustering results were obtained by selecting different sets of the isoforms/genes with the greatest variation (see Additional file 3: Figures S1 and S2).

We expected the clustering of samples to result in a first-level grouping of different tissues, followed by a second-level grouping of cancer and non-oncogenic cell lines within each tissue type. Unexpectedly, however, we found almost uniform grouping of cancer and non-oncogenic cell lines into two large clusters, with an overall cluster purity of 97.5% at isoform level (Figure 1A). Further, the samples belonging to same cell/tissue type within each cancer/non-oncogenic group were clustered together, confirming the discriminatory power of isoform level gene-expression profiles. For example, the paired non-oncogenic melanocyte and cancerous melanoma samples, and the matched pairs of MCF7 (breast-cancer cell line) and the HMEC samples (non-oncogenic origin) were separated correctly into either non-oncogenic or cancer groups, with one exception from MCF7 samples (Figure 1A). Overall, only four samples, two each from non-oncogenic and cancer cell lines, were clustered into the wrong group. The cancer cell lines that were clustered into the non-oncogenic group were one MCF7/mammary gland adenocarcinoma (GEO accession number GSM472936) and one pancreatic carcinoma cell line (GEO GSM472939), and the non-oncogenic samples that were assigned to the cancer group were one adult non-oncogenic human epidermal keratinocyte (NHEK) sample (GEO GSM472937) and one non-oncogenic umbilical vein endothelial cell (HUVEC) sample (GEO GSM472935).

Although the clustering at gene-level showed some power of discrimination between non-oncogenic and cancer cell lines, the overall grouping was significantly less efficient than the clustering at isoform-level. The gene level cluster purity was 75%, with 20 samples from the non-oncogenic and cancer cell lines clustered into the wrong group (Figure 1B). The better separation of non-oncogenic and cancer cell lines at isoform-level (97.5% cluster purity) compared with gene -(75% cluster purity) implies that gene-expression profiles in cancer cells are more specifically altered at isoform-level for numerous genes, which could not be detected using gene level analysis.

**Figure 2:**
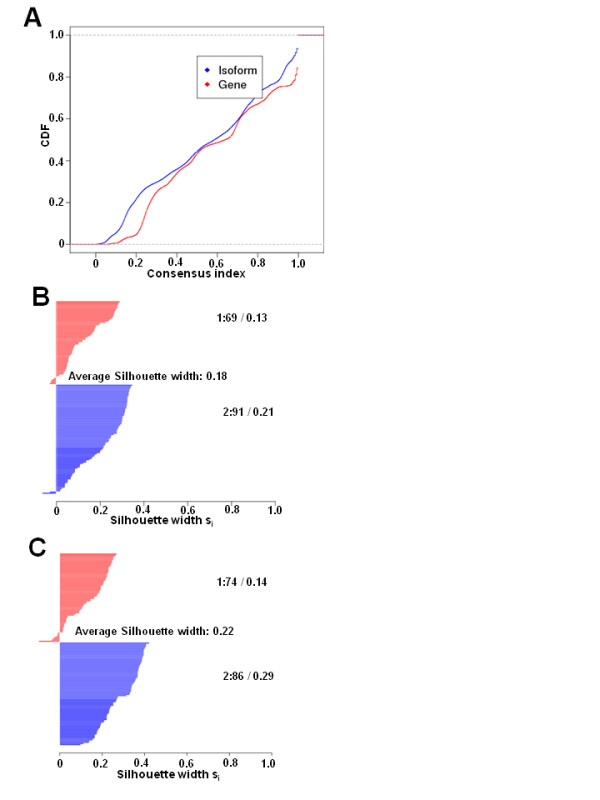
**Cumulative distribution function (CDF) and silhouette width plot demonstrates that isoform level clustering is more robust than gene-level clustering**. **(A) **The empirical CDF plots were based on resampling 200 times at either isoform or gene level. **(B, C) **Silhouette width plots of the clustering results based on **(B) **gene-level expression of 27,063 genes and **(C) **isoform-level expression of 87,345 transcripts for the 160 datasets representing oncogenic and non-oncogenic cell lines. The stability and robustness of the clusters is indicated by average silhouette width. The sample number falling in each cluster and the silhouette width of each cluster is also represented on the figure.

We also appliedconsensus hierarchical clustering to compare the stability of the isoform-based approach to the gene-based approach [33,38]. The empirical cumulative distribution function (CDF) plot of the consensus index (Figure 2A) indicated that isoform-based clustering gives more stable results than gene-based clustering. We further plotted the silhouette width for isoform-based and gene-based clustering (Figure 2B and 2C, respectively) [39]. The larger silhouette width of one sample indicates higher similarity to its own group than to any other group member. The average silhouette width for isoform-based clustering was 0.22, which was larger than the gene-based width of 0.18, indicating that the clustering based on isoforms is more homogenous than that based on genes.

We next focused our analysis on two specific cancers, breast cancer, and melanoma, for which we had matched oncogenic and non-oncogenic cell lines, in addition to the comparison of all oncogenic versus all non-oncogenic cell lines.

### Transcript variants of numerous genes were differentially expressed between non-oncogenic and cancer cell lines

**Figure 3:**
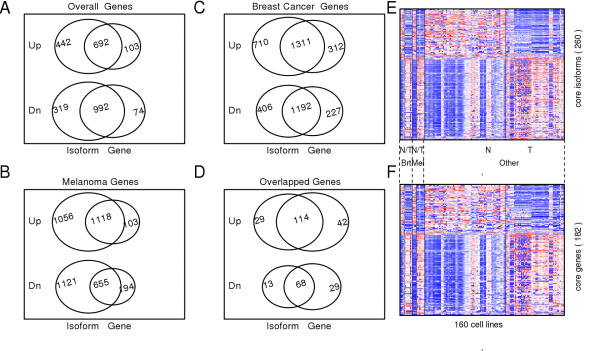
**Venn diagram showing the overlapping genes that were significantly upregulated (Up) or downregulated (Dn) either at gene level or isoform level in cancer**. If more than one isoform from a single gene were significantly upregulated or downregulated, they were counted only once in the diagram. The differentially expressed genes/transcript variants were obtained by comparing the groups of **(A) **all non-oncogenic and all oncogenic cell lines, **(B) **melanocyte and melanoma cell lines and **(C) **human mammary epithelial cell (HMEC) and human breast adenocarcinoma (MCF7) cell lines. **(D) **Venn diagram shows the overlap of genes in A, B and C. The overlapping genes that were either upregulated (114 genes) or downregulated (68 genes) both at isoform and gene level were designated as the core isoforms and core genes. The 260 isoforms correspond to 182 unique genes. **(E, F) **Heat-map diagrams of differential gene expression of **(E) **core isoforms and **(F) **core genes.

We evaluated differential gene expression, both at gene and isoform level, between 1) all non-oncogenic and all cancer cell lines (see Additional file 4, Table S3), 2) a non-oncogenic breast cell-line (HMEC) and a breast-cancer cell-line (MCF7)cell lines (see Additional file 5: Table S4), and 3) non-oncogenic melanocytes and melanoma cell lines (see Additional file 6: Table S5) (Figure 3A-C). After performing the three comparisons independently, we overlapped the identified gene sets to identify those genes or gene isoforms that were consistently upregulated or downregulated in cancer compared with non-oncogenic cell lines (see Additional file 7, Table S6). We denoted the genes that were found to be significantly different between non-oncogenic and cancer groups in all the three comparisons as the core set of genes/gene isoforms (Figure 3D). Interestingly, we found numerous genes that were significantly differentially expressed at isoform level but not at gene level. A gene was declared as differentially expressed at isoform level if at least one of its isoforms showed significant differential expression between non-oncogenic and cancer groups. For example, 29 and 13 genes were found to be significantly upregulated and downregulated, respectively, at isoform level but not at gene-level in all three pairwise comparisons (Figure 3D). Overall, we found a total of 260 different transcript variants or gene isoforms (Figure 3E) of 182 unique genes (Figure 3F) that had significant gene-expression differences at either isoform or gene level.

**Figure 4:**
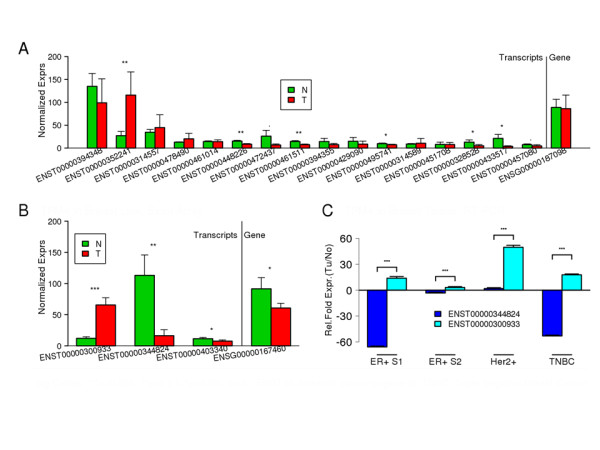
**Mean normalized expression estimates of *MITF *and its transcript variants in melanocyte (N) and melanoma (T) cell lines, and of *TPM4 *and its transcript variants in non-oncogenic and oncogenic breast cell lines (HMEC-N; MCF7-T) and tissues**. **(A) **Notice that although the gene level expression of *MITF *was not significantly different between melanoma and melanocytes, three of its isoforms showed significant differences in their expression. Whereas ENST00000352241 was overexpressed, ENST00000433517 and ENST00000472437 were underexpressed in melanoma. **(B) **Similarly, two transcript variants of *TPM4 *showed opposing expression patterns between the non-oncogenic HMEC and the MCF7 breast-cancer cell lines. **(C) **Validation results via RT-qPCR experiments showing the relative fold expressions of the two major transcript variants of *TMP4 *(ENST00000300933 and ENST00000344824) in various human breast-cancer tissue subtypes compared with the surrounding matched non-oncogenic breast tissue. We analyzed two patient samples for estrogen receptor (ER)-positive breast-cancer tissues (ER+S1 and ER+S2) and one patient sample each for Her2 gene-positive breast cancer (Her2+) and triple-negative breast cancer (TNBC) subtypes. MCF7 is an ER+ breast-cancer cell line.

In each pair of comparisons, of the total genes identified to be significant at isoform level, at least 30% (range from 30% to 55%) were found to be significant only at isoform level. In other words, more than one isoforms of these genes displayed differential expression between non-oncogenic and cancer samples, but the gene-level expression differences were cancelled out by the combined effect of various isoforms of the same gene. These genes displayed alternate splicing between non-oncogenic and cancer cell lines. This observation strongly supports previous reports such as that by David and Manley [18]. For example, the *MITF *(micro-ophthalmia transcription factor) gene uses at least nine different promoters and first exons to generate a remarkably diverse set of mRNAs and protein isoforms that differ at the N-terminus. The gene platform we used (Affymetrix GeneChip Human Exon 1.0 ST Array) has probe sets corresponding to 16 different transcript variants of this gene, based on Ensembl gene annotations. The alternative promoters of *MITF *reflect the tissue specificity of its isoforms, which are selectively expressed in melanocytes, macrophages, osteoclasts, heart muscle, or retinal pigmented epithelium. *MITF*, generally believed to play a primary role in melanocyte stem-cell proliferation and expression of a set of pigment-related genes [40], has been shown to be amplified in a small percentage (10 to 20%) of melanomas, and seems to confer a poor prognosis when overexpressed [41]. In the comparative analysis between non-oncogenic melanocytes and melanoma cell lines (Figure 3C), no differential expression of *MITF *was found by the gene level analysis. However, the *MITF *isoform ENST00000352241 was found to be significantly overexpressed in melanoma compared with melanocytes (FC = 3.4), whereas the transcript variants ENST00000433517 (FC = -5.6) and ENST00000472437 (FC = -3.4) were underexpressed in melanoma (Figure 4A). Similarly, the *TPM4 *gene was seen to have weak differential expression in MCF7 compared with HMEC cell lines samples. However, although one of the *TPM4 *isoforms (ENST0000030933) was found to be strongly overexpressed (FC = 5.47), another isoform (ENST00000344824) was found to be significantly underexpressed in MCF7 samples (FC = -7.75) (Figure 4B). These two isoforms thus cancelled each other out, resulting in the overall gene expression not being significantly different between the non-oncogenic and oncogenic cell lines. *TPM4 *has been reported to be differentially expressed in breast cancer [42]. Our analysis suggests that whereas gene level expression estimates of *TPM4 *and *MITF *contribute little to the discrimination of cancer cell lines from non-oncogenic cells, expression estimates specific to one or more isoforms of these genes have a better discriminating power. Interestingly, we found a total of 294 isoforms, corresponding to 110 genes in melanoma (see Additional file 6: Table S5), and 75 transcript isoforms, corresponding to 16 genes in breast cancer (see Additional file 5: Table S4), that showed opposing expression patterns at isoform level.

### Experimental validation of differentially expressed transcript variants in breastcancer cell lines and samples

To validate the existence of the opposing expression patterns of isoforms for various genes, we measured isoform expression by RT-qPCR for two opposing isoforms of seven genes in three breastcancer cell lines relative to the non-oncogenic HMEC cell line (Tables 1). The expression pattern of isoforms obtained from exon-array and RT-qPCR experiments were similar for all seven genes in MCF7 cell lines. However, in the case of MDA-MB-231 and T47D cell lines isoforms of four and two of the seven genes, respectively had an expression pattern similar to that seen in the exon-array data for MCF7. To further validate opposing transcript expression in patient samples between non-oncogenic and cancer tissues, we selected the *TPM4 *gene in breast cancer as an example. The opposing expression patterns of the *TPM4 *isoforms were confirmed in the estrogen receptor-positive and triple-negative breast cancer sample. Although the Her2+ sample did not show the opposing pattern of expression, one isoform had the significantly highest fold change of all the samples (Figure 4C). In all samples, the simple Student *t*-test results between the averaged fold expressions of the two isoforms were all significant (*P *< 0.001). These results strongly support our hypothesis that isoforms of multi-transcript genes can have opposing roles in cancer.

**Table 1:** Isoform expression in breast-cancer cell lines as measured by reverse transcriptase -quantitative PCR (RT-qPCR).

			RT-qPCR		
			
Gene	Transcript ID	Exon array	MCF7	MDA-MB-231	T47D
*TPM4*	ENST00000344824	-7.75	-500	-5.55	-111.11
	
	ENST00000300933	5.47	2.14	1.01	-2.08

*WDR45*	ENST00000460501	2.86	5.22	2.17	1.24
	
	ENST00000486337	-2.48	-2.22	-1.61	1.12

*GART*	ENST00000381831	2.38	1.66	-1.42	2.78
	
	ENST00000381815	-3.83	-2.17	-1.13	1.09

*FLII*	ENST00000474265	-3.48	-2.32	-1.58	1.58
	
	ENST00000461110	2.27	1.27	1.85	3.14

*CHN1*	ENST00000490654	2.47	109	193	12
	
	ENST00000444573	-4.27	-16.66	10.23	-55.55

*OXR1*	ENST00000312046	3.72	19.7	33.5	13.6
	
	ENST00000445937	-3.6	-100	3.37	-26.31

*SRGAP3*	ENST00000489616	2.11	3.61	2.88	12.93
	
	ENST00000475560	-4.39	-12.5	-500	1.04

The fold expression of the indicated transcripts in the three cell lines was calculated relative to HMEC (N) cells.

### Supervised analysis identified an isoform set able to separate the tumor lines from normal lines in an almost perfect pattern

**Figure 5:**
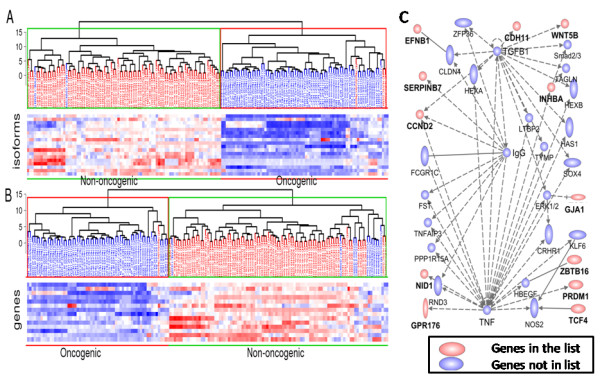
**Hierarchical clustering dendrograms and heat map based on core transcript and gene expression and the gene network for the core genes**. The hierarchical clustering dendogram of 160 samples and heat map of gene-expression estimates of (A) 18 core transcripts and (B) 14 core genes that were significantly enriched in the top 5 gene networks identified by Ingenuity Pathway Analysis (IPA). The 18 transcripts were those having the maximum coefficient of variation (CV) values of all the core isoforms, representing 14 core genes. (**C**) IPA gene network of the 14 core genes, of which 12 belong to the canonical pathway with associated known functions in hematological system development and function, tissue morphology, and cellular development (in red).

We performed IPA (version 6.0; Ingenuity^® ^Systems, Redwood City, CA, USA) [36] to find significant molecular functional categories enriched in the differentially expressed gene sets, and transformed the target genes into a set of relevant networks by using literature-based records that are maintained in the IPA Base. We first performed this analysis using the core gene set of 182 genes that were consistently up-regulated or downregulated in cancer cell lines (Figure 3F). The analyses found 10 molecular and cellular functions that were significantly enriched in the core gene set (see Additional file 8: Table S7). Interestingly, the top five molecular and cellular functions identified by IPA were 'Role of BRCA1 in DNA damage response', 'Mitotic roles of Polo-like kinase', 'Hereditary breast cancer signaling', 'Role of CHK proteins in cell cycle checkpoint control', and 'Cell cycle: G2/M DNA damage checkpoint regulation', which are frequently deregulated in cancer initiation and progression. Therefore, we considered that the core genes involved in these pathways might also be useful to effectively separate cancer from non-oncogenic samples. To test this hypothesis, we repeated the clustering analysis, using the core genes and their isoforms that belonged to the five most significant pathways (Figure 5). The clustering analysis performed by filtering out the isoforms for which there was relatively little variation in expression estimates across all the samples produced an almost identical result (18 isoforms, corresponding to 14 unique genes, Figure 5A) to that obtained by using all the gene isoforms (Figure 1A). However, at gene level, the clustering based on these 14 genes produced a comparable result, but with relatively lower cluster purity (92.5%, or 12 of 160 cell lines were grouped in the wrong cluster) than at isoform level (97.5% or 4 of 160 were grouped in the wrong cluster) (Figure 5B).

These 14 genes are *WNT5B, CCND2, SERPINB7, GPR176, INHBA, EFNB1, PTRF, CDH11, ZBTB16, GJA1, COL5A2, NID1, PRDM1*, and *TCF4*. Except for *CCND2 *and *GPR176*, all other genes in our database have more than one isoform. Four genes (*SERPINB7, INHBA, GJA1*, and *NID1*) have two isoforms that have significantly different expression between the cancer and non-oncogenic groups. Interestingly, 12 of these 14 genes belong to the same gene network, involved in hematological system development and function, tissue morphology, and cellular development. According to the Ingenuity Pathway Knowledge Base, the network consists of a total of 27 different genes, which suggests that almost 50% of the genes belonging to this network are dysregulated either at the gene or isoform level between non-oncogenic and cancer cells (Figure 5C). Moreover, most of these genes have already been implicated both in tumorigenesis and in several developmental processes [43-48]. For example, it was shown that the phosphorylation-dependent interaction between c-Jun and TCF4 regulates intestinal tumorigenesis by integrating c-Jun kinase (JNK) and adenomatous polyposis coli (APC)/β-catenin, two distinct pathways activated by Wnt signaling [49]. Multiple alternatively spliced transcript variants that may encode different protein isoforms of these genes (for example, *TCF4, WNT5B*) have been described. Therefore, it would be interesting to evaluate the components of this gene network in different cancers at isoform level.

### Gene level and isoform level analyses identified interesting pathways associated with cancer

**Figure 6:**
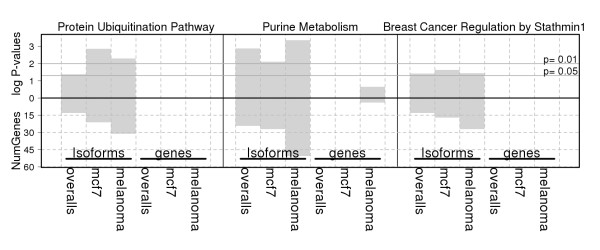
**Canonical pathways significantly enriched with genes that are differentially expressed at isoform level but not at gene level in three pairwise comparisons**. 1) Overall: all oncogenic versus all non-oncogenic cell lines, 2) mcf: MCF7 versus HMEC, and 3) melanoma: melanoma cell -lines versus melanocytes. Number of genes and the log *P *values for each pathway are plotted in the lower and upper panels of the bar chart, respectively.

To test whether the genes that are differentially expressed at isoform level but not at gene level could reveal interesting pathways associated with cancer, we focused the pathway-enrichment analysis on two different gene sets: 1) genes that are significant at isoform level only and 2) genes that are significant at gene level only Figure 3A-C; genes without overlaps in the middle). We performed this analysis separately for each of the three comparisons (all cancer versus all non-oncogenic; HMEC versus MCF7; and melanocytes versus melanoma cell- lines) between the non-oncogenic and cancer pairs. This analysis led to the identification of three canonical pathways (protein ubiquitination, purine metabolism, and breast-cancer regulation by stathmin1) that were significantly enriched in isoform level gene sets, but not in gene level gene sets (Figure 6).

## Discussion

Human cancer is a complex disease. It is known that most of the genes in mammalian genomes generate different transcript variants and protein isoforms, which often function in a cell/tissue type-specific and developmental stage-specific manner in non-oncogenic cells. Cancer results from the sequential acquisition of a number of genetic and epigenetic alterations, and these mutations may alter the expression of specific isoforms but not the others of a gene. Despite this growing knowledge, most biomarker and drug-discovery studies still evaluate expression differences and study gene regulatory mechanisms at gene level rather than at isoform level. In this study, we have shown that oncogenic cell lines could be more accurately discriminated from non-oncogenic cell lines, regardless of their cells of origin, by gene-expression profiling at isoform level compared with gene level. In spite of the differences in tissues of origin, the cell lines were broadly clustered into two groups, non-oncogenic and oncogenic, by isoform level gene-expression profiles. We noted that numerous genes showed differential expression at isoform level but not at gene level. For some of these genes, the differential expression of alternative transcripts occurred in the opposite direction; while some of the isoforms of the same gene were upregulated, others were downregulated, resulting in them cancelling each other out and producing insignificant expression differences at gene level between cancer and non-oncogenic groups. Our findings are in agreement with a previous study on prostate cancer that investigated the expression of 1532 splice forms for 364 prostate cancer-related genes, using data from a customized exon junction array [20]. The authors found that many genes were differentially expressed at splice-form level but not at gene level and this increase in the number of differentially expressed variables at splice-form level contributed to a 92% accuracy for a 128 splice-form-based classifier for normal and cancerous prostate tissue, whereas the accuracy was 87% using a classifier based on 32 genes. That study profiled 1532 mRNA splice forms from 364 potential prostate cancer-related genes, whereas in the current study, we used genome-wide exon-array data that identified the expression of 114,930 transcripts/isoforms corresponding to 35,612 different genes, including all known non-coding genes in the Ensembl database. In addition, our study focused on discriminating oncogenic and non-oncogenic cells in general, irrespective of their tissue of origin. Using genome-wide isoform level versus gene-level expression information, we found that oncogenic and non-oncogenic cells could be segregated using isoform level information with 97.5% accuracy versus 75% accuracy for gene level information, and even a smaller signature of 18 isoforms was effective in separating the two groups, with equal accuracy. These subtle differences at isoform level in discriminating non-oncogenic and oncogenic cell lines reflect the fact that gene level expression measurements, whose estimates are generally the summation of all the isoform level expression estimates of individual genes, are less accurate in characterizing cancer and non-oncogenic cells.

The pathway-enrichment analysis of genes that are differentially expressed in cancer cell lines at isoform level but not at gene level produced three interesting pathways that have been implicated in various cancers. It is well known that protein phosphorylation and protein ubiquitination regulate most aspects of cell life, and defects in these control mechanisms cause cancer and many other diseases [50]. Similarly, abnormalities in purine metabolism and over-expression of *Stathmin 1 *(*STMN1*) are characteristic features of many human tumors [51,52]. The key genes of these pathways (for example, *STMN1, PNP, RPS27A *and *UBA52*) transcribe different transcript variants, some of which encode different protein isoforms. It is therefore necessary to evaluate the gene-expression differences and to study gene regulatory mechanisms at isoform level rather than at gene level between non-oncogenic and disease conditions, such as cancer. Recent advances in cancer genomics have shown that gene-expression signatures are useful for biomarker identification and drug discovery [53]. In this regard, the present study highlights the importance of studying gene-expression signatures at isoform level rather than at gene level, and makes a strong case for isoform level gene/protein-expression profiling methods for improved cancer biomarker and therapeutic discovery.

## Conclusions

In conclusion, we have identified a common, isoform level signature that can be used to discriminate effectively between cancer and non-cancer cell lines. We found numerous genes for which the differential expression of alternative transcripts occurred in opposing directions, with some of the isoforms of the same gene being upregulated while others were downregulated, resulting in insignificant expression differences at gene level between cancer and non-oncogenic groups. This is supported by our experimental validation of opposing expression patterns for *TPM4 *gene isoforms in non-oncogenic and oncogenic tissue samples from breast cancer patients. The present study highlights the importance of studying gene-expression signatures at isoform level rather than at gene level in characterizing the cancer transcriptome.

## Competing interests

The authors declare that they have no competing interests.

## Authors' contributions

ZZ designed the computational methods and performed the statistical analyses. SP designed the experimental methods and performed the RT-qPCR experiments. YB performed statistical analyses. JT participated in the design of the study, and provided non-oncogenic breast and oncogenic breast tissue samples. RD and JT formulated and directed the design of the study. ZZ, SP, and RD wrote and edited the manuscript. All authors read and approved the final manuscript.

## Supplementary Material

Additional file 1 **Supplementary Table S1**. Summary of cell lines samples and the exon-array datasets used in this study. Excel document. Click here for file

Additional file 2 **Supplementary methods and Table S2 A and B**. Table S2A Clinical characteristics of samples from patients with breast cancer used in this study. Table S2B Primer sequence used for real-time quantitative reverse transcriptase (qRT)-PCR to measure isoform expression. Word document. Click here for file

Additional file 3 **Supplementary Figure S1 and S2**. Dendrograms representing hierarchical clustering of 160 (73 oncogenic and 87 non-oncogenic) datasets from cell lines using expression estimates of (Figure **S1**) transcripts and (Figure **S2**) genes at different cut-off points for coefficient of variation (CV). Powerpoint document. Click here for file

Additional file 4 **Supplementary Table S3**. List of genes and isoforms differentially expressed between the oncogenic and non-oncogenic cell lines groups (Figure 3A). Excel document. Click here for file

Additional file 5 **Supplementary Table S4**. List of genes and isoforms differentially expressed between non-oncogenic HMEC and MCF7 breast-cancer cell lines (Figure 3C). Excel document. Click here for file

Additional file 6 **Supplementary Table S5**. List of genes and isoforms differentially expressed between the melanocyte and melanoma cell lines (Figure 3B). Excel document. Click here for file

Additional file 7 **Supplementary Table S6**. List of overlapped genes and isoforms consistently upregulated or dow regulated across all three pairs of comparisons (Figure 3D). Excel document. Click here for file

Additional file 8 **Supplementary Table S7**. Pathways associated with the top gene networks identified by IPA analysis of core genes. Word document. Click here for file
