# Supplementary material for: Isoform level expression profiles provide better cancer signatures than gene level expression profiles
Source: Genome Med. 2013 Apr 17;5(4):33. doi: 10.1186/gm437 (PMC3706752; doi:10.1186/gm437)
Supplement: Additional file 2 — Supplementary methods and Table S2 A and B. Table S2A Clinical characteristics of samples from patients with breast cancer used in this study. Table S2B Primer sequence used for real-time quantitative reverse transcriptase (qRT)-PCR to measure isoform expression. Word document. [file gm437-S2.DOCX]

**Isoform level gene expression signature is a better discriminator of oncogenic from non-oncogenic cell lines than gene level expression signature**

ZhongFa Zhang et al

**Supplementary Materials and Methods in Details**

**Raw Data Process** We downloaded raw data sets from NCBI public data depository (GSE15805(1), GSE17778, GSE19090(2) and GSE17349 (3)). The first three GSEs were deposited by the Encode project, while the last data set was deposited by an independent study project (3). The data sets contain 79, 36, 83 and 8 cases of various types of cell lines using Affymetrix exon array platform (so the total adds up to 206 cases). There are 10 samples appeared in both GSE15805 and GSE17778 datasets, 2 stem cell lines and 32 cell lines originated from blood or blood related, such as B-cell, T-cell, or lymphoblastoma. Our profiling study revealed that these blood (or its related) and stem cell samples were quite different in expression profile from the samples of solid tissue origins. They are hard to be defined non-controversially as either oncogenic or normal. There are 2 progeria fibroblast samples. Progeria is an extremely rare genetic condition (about 1 in 4 - 8 million newborns, see <http://www.progeriaresearch.org/>) wherein symptoms resembling aspects of aging are manifested at an early age. It is neither an tumor condition nor a normal condition. We decided to exclude the above samples (blood related, progeria fibroblast and stem cells) from our analysis. We now have 160 samples left for our analysis. Of them, there are 8 melanoma samples and 4 normal melanocyte samples as our first matched normal and tumoral pair and 4 normal human mammary epithelial cells (HMEC) and 8 human breast adenocarcinoma cell line (MCF7) as our second matched normal and tumoral pair. A cell line will be classified as tumoral (oncogenic) if it is oncogenic when it is implanted in living cells. Otherwise, it is classified as normal (or non-oncogenic). In this paper, we refer a cell line as tumor or normal based on this classification. The complete classification and labeling information of cell lines used in this study were summarized in Tables 1 and 2.

**The gene expression and isoform expression determination** The raw exon array data was processed by the Multi-Mapping Bayesian Gene eXpression (mmbgx) algorithm for Affymetrix whole-transcript arrays (4). Briefly, the probe level data of CEL files was read into the system and a Bayesian model was used to calculate the isoform expressions as well as the gene expressions based on Ensemble database (version 56, (5-6)). We set the burn-in iteration to be 4096 and real iteration to be 8096, instead of their default values of 8096 and 16192 respectively at both gene and isoform levels, as the time required for each sample is a little long. The algorithm gives good estimation of both gene expressions and isoform expressions (see Supplementary Figure S5 for the stability study of the algorithm). For example, 2 independent runs on the same sample give almost identical expression levels while runs on the different samples give comparable results, but much less correlated than that on the same sample. The algorithm has been verified on mouse tissues at isoform level through RT-PCR(4).

The raw expression levels were normalized using the locally weighted scatter plot smoothing (*loess)* algorithm (7-8), also implemented in the mmbgx algorithm. This generated two data sets, one for transcript expression (114930 transcripts and 160 samples) and another for gene expression data (35612 unique genes and 160 samples). Incidentally, there are a very number of transcripts in the output data set which are not unique in terms of sequence identities. However, we decide not to filter them out in our analysis, as their existence in the data set causes no troubles in our later profiling analyses. Two isoforms could be joined together, due to the uncertainty of their uniqueness as distinguishable isoforms due to lack of enough information.

**Gene and transcript filtering algorithm** We used unsupervised clustering algorithm implemented in R to study the profile similarity between the samples in the study cohort at both gene and isoform levels on the properly filtered data sets. The filtering algorithm was based on the coefficient of variation (CV) quantity, defined to be the standard deviation adjusted (divided) by the mean value when the expressions are expressed in log scale. The coefficient of variation is useful because the standard deviation of expression intensities must always be understood in the context of the mean value of the expressions. Unlike standard deviation, which was heavily affected by the mean value of the data set, CV is a dimensionless number, is a way to penalize the expressions with overall high expression values. The CVs for all isoforms are calculated and the quantiles of the CVs are calculated too. Isoforms with CV values smaller than the p-th quantile are dropped off (so the cutoff value is p). We used a sequence of cutoff values from 0.1 to 0.8 to filter the isoforms. The isoforms passed the filtering criteria are those representing the most varied ones among all isoforms in the genome (after adjustment of the mean expression levels). Similar filtering process was applied to the expression data at gene level to obtain the filtered gene expressions before the clustering algorithm was applied.

**Clustering algorithm**

We used the general hierarchical cluster algorithm to cluster the samples (9). More specifically, we used Euclidean distance as a measurement for dissimilarities. To find the differential genes between two conditions, we used *limma* method (10-11). An isoform or gene was selected if both its fold change is greater than a cutoff value and the FDR adjusted p-value is smaller than a cutoff value are satisfied. In our study, we used 2 for fold change cutoff and 0.01 as cutoff for the adjusted p-values for all comparisons between two conditions. Ingenuity Pathway Analysis (IPA, http://www.ingenuity.com/) was used to associate the identified gene sets with biological functions, canonical pathways and networks. The canonical pathway refers to pathways in the IPA data base collected over time by the company. To identify pathway differences arising from gene sets identified at either isoform level or at gene level, we used the counting method on the p-values of pathways from IPA analysis; the p-values were used as an indicator of association strength between the gene sets and pathways. Among the three pairwise tumor/normal comparisons, a pathway will be selected and reported if it is found to be significantly associated with the gene sets identified at isoform level in all three pairs of comparisons, but to be not significantly associated with the gene sets identified at gene level at gene level in all three pairs of comparisons, or vice visa. The significance level was set to be 0.05 in all comparisons.

All calculations were based on Bioconductor version 2.8 or above and R platform of version 2.10 (12).

**References**

1. McDaniell R, Lee BK, Song L, Liu Z, Boyle AP, Erdos MR, et al. Heritable individual-specific and allele-specific chromatin signatures in humans. Science. 2010;328:235-9.

2. Hansen RS, Thomas S, Sandstrom R, Canfield TK, Thurman RE, Weaver M, et al. Sequencing newly replicated DNA reveals widespread plasticity in human replication timing. Proc Natl Acad Sci U S A. 2010;107:139-44.

3. Berger MF, Levin JZ, Vijayendran K, Sivachenko A, Adiconis X, Maguire J, et al. Integrative analysis of the melanoma transcriptome. Genome Res. 2010;20:413-27.

4. Turro E, Lewin A, Rose A, Dallman MJ, Richardson S. MMBGX: a method for estimating expression at the isoform level and detecting differential splicing using whole-transcript Affymetrix arrays. Nucleic Acids Res. 2010;38:e4.

5. Yates T, Okoniewski MJ, Miller CJ. X:Map: annotation and visualization of genome structure for Affymetrix exon array analysis. Nucleic Acids Res. 2008;36:D780-6.

6. Okoniewski MJ, Yates T, Dibben S, Miller CJ. An annotation infrastructure for the analysis and interpretation of Affymetrix exon array data. Genome Biol. 2007;8:R79.

7. Cleveland WS, Grosse E, Shyu WM. Local regression models: Wadsworth & Brooks/Cole; 1992.

8. Cleveland WS. Robust Locally Weighted Regression and Smoothing Scatterplots. Journal of the American Statistical Association. 1979;74:829-36.

9. Gordon ADSE. Classification. London: Chapman and Hall / CRC; 1999.

10. Smyth GK, Yang YH, Speed T. Statistical issues in cDNA microarray data analysis. Methods Mol Biol. 2003;224:111-36.

11. Smyth GK. Linear models and better empirical bayes methods for assessing differential expression in microarray experiments. Stat Appl Genet Mol Biol. 2004;3:Article3.

12. R Development Core Team. R: A Language and Environment for Statistical Computing, R Foundation for Statistical Computing. . Vienna, Austria. <http://www.R-project.org;> 2010.

**Supplementary table S2A: Clinical characteristics of the patient samples used for measuring *TPM4* expression in primary tumor tissues.**

| ID | Race | Disease status | Age at diagnosis | Type | Tumor size (cm) | Grade | No. of (+) axilla nodes | ER (%) | PR (%) | Her2 (%) | FISH |
| --- | --- | --- | --- | --- | --- | --- | --- | --- | --- | --- | --- |
| **TB145** | **hispanic** | **NED** | **46** | **IDC** | **4.5** | **3** | **3** | **80** | **40** | **2+, 30%** | **+ve** |
| **TB146** | **caucasian** | **Deceased** | **40** | **IDC** | **11.6** | **3** | **4** | **60** | **80** | **0** | **n.d.** |
| **TB147** | **caucasian** | **NED** | **64** | **IDC** | **2.3** | **3** | **0** | **0** | **0** | **0** | **-** |
| **TB149** | **caucasian** | **NED** | **71** | **IDC** | **2.3** | **2** | **3** | **95** | **95** | **0** | **n.d.** |

**Supplementary table S2B: Primer sequence used for RT-qPCR experiments**.

| Gene | Transcript Id | Primer sequence (5'--3') |
| --- | --- | --- |
| *Tpm4* | ENST00000344824 | Forward: AGCCATGGAGGCCATCAA |
|  |  | Reverse:TCCTCAGCGGCTTTCTTATCC |
|  | ENST00000300933 | Forward:GGTGAAACGCAAGATCCAG |
|  |  | Reverse:CATCACCTTCAGCTTTCTCG |
| *WDR45* | ENST00000460501 | Forward:CAAGCCGGTCCAACGTTT |
|  |  | Reverse:GCCAAAGGCCCTGACACTTA |
|  | ENST00000486337 | Forward:TGGAGCCTGGCGAGCTT |
|  |  | Reverse:CGAAGGCGCAGATGCAA |
| *GART* | ENST00000381831 | Forward:GGCTTGCTAAGTGCCTGAGATT |
|  |  | Reverse:CGGGTTGCCCTTCTTGCT |
|  | ENST00000381815 | Forward: ggtgtcggtttcattttcct |
|  |  | Reverse: GCTGCCATtgttctgtctgt |
| *FLII* | ENST00000474265 | Forward:CCGGTGCTCCAACGAGAA |
|  |  | Reverse:CTTGGCAAAAGTCGGAGCAT |
|  | ENST00000461110 | Forward:CCCGCAACCCCCATGT |
|  |  | Reverse:GCTGTGCGCCCAAGTTTC |
| *CHN1* | ENST00000490654 | Forward:TCCATCCACGATCTGGTGACT |
|  |  | Reverse:TTCTGCTGCCTTGGTTTCAA |
|  | ENST00000444573 | Forward:TGGTCAGGGAGGAAAACTAATAGG |
|  |  | Reverse:TGCTGACGGCCCTCTTGT |
| *OXR1* | ENST00000312046 | Forward:GGACTACCTGACGACGTTCA |
|  |  | Reverse:GCTCCCTCAGGTAATCTTCG |
|  | ENST00000445937 | Forward:AAAGTCCCAGTCGGTGGATA |
|  |  | Reverse:GCTGCATTGTTCTGCTCTTC |
| *SRGAP3* | ENST00000489616 | Forward: tgctttttagggatgagcaa |
|  |  | Reverse: tgcttacgcaacggataaag |
|  | ENST00000475560 | Forward: cctaggaccatggaaaagga |
|  |  | Reverse: gcagcttggtgatgaggtta |
