# Supplementary figures and images for: Isoform level expression profiles provide better cancer signatures than gene level expression profiles
Source: Genome Med. 2013 Apr 17;5(4):33. doi: 10.1186/gm437 (PMC3706752; doi:10.1186/gm437)

## Slide 1
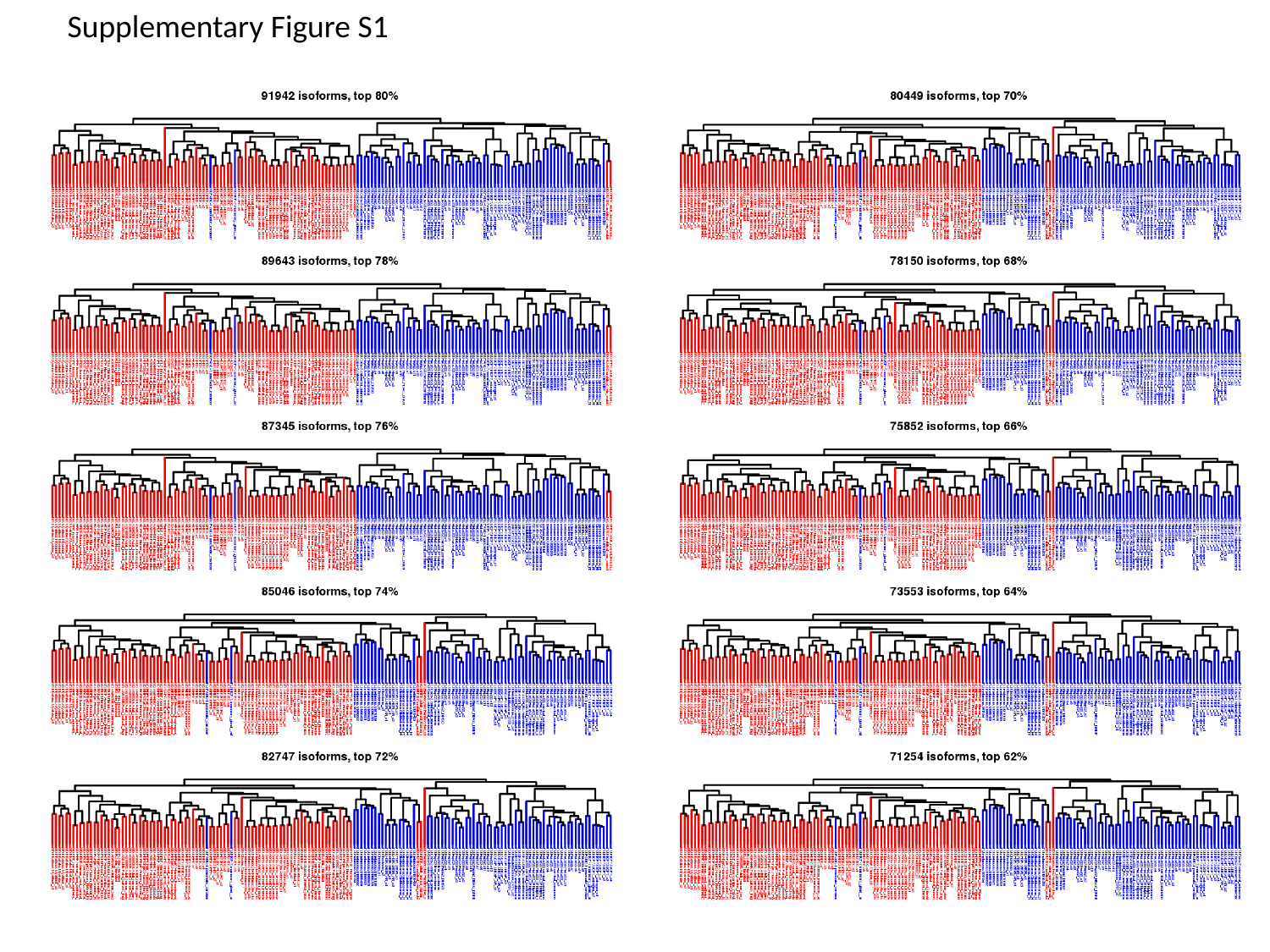

Supplementary Figure S1

## Slide 2
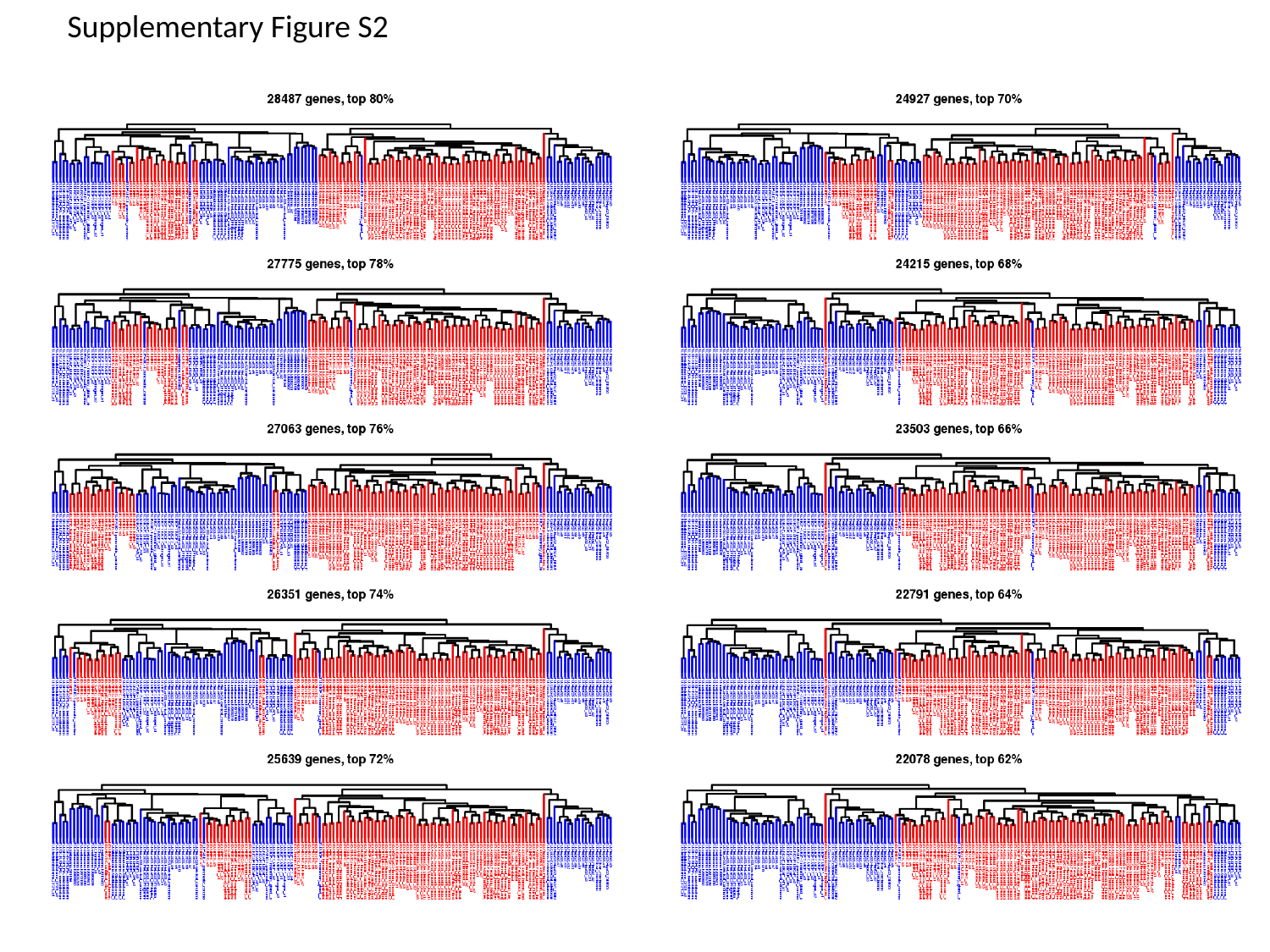

Supplementary Figure S2

Supplement: Additional file 3 — Supplementary Figure S1 and S2. Dendrograms representing hierarchical clustering of 160 (73 oncogenic and 87 non-oncogenic) datasets from cell lines using expression estimates of (Figure S1) transcripts and (Figure S2) genes at different cut-off points for coefficient of variation (CV). Powerpoint document. [file gm437-S3.PPTX]
