# Supplementary material for: Isoform level expression profiles provide better cancer signatures than gene level expression profiles
Source: Genome Med. 2013 Apr 17;5(4):33. doi: 10.1186/gm437 (PMC3706752; doi:10.1186/gm437)
Supplement: Additional file 8 — Supplementary Table S7. Pathways associated with the top gene networks identified by IPA analysis of core genes. Word document. [file gm437-S8.DOCX]

**Supplementary table S7: Pathways associated with the top gene networks identified by IPA analysis of core genes.**

| **Top functions** | **Score** | **Number of genes in list** | **Genes in network (genes identified are marked in bold)** |
| --- | --- | --- | --- |
| Cell Cycle, Cellular Assembly and Organization, DNA Replication, Recombination, and Repair | 51 | 31 | **ATAD2, BIRC5, BLM, BRCA2, C13ORF15, CCNA2, CDC25C, CDCA8, CDK1, CDT1, CIT,** Cyclin A, E2f, **E2F1, ERCC6L, FANCA, FANCD2, FEN1, GJA1, HIST1H3B (includes EG:8358),** Hsp90, **KIF14, KIF4A, KIFC1, MCM10, MYBL2, NUSAP1, PLK1, PRC1, PRR11,** RAD51L3**, TK1, TTK, UBE2C, XRCC2** |
| Cell Cycle, DNA Replication, Recombination, and Repair, Cancer | 19 | 16 | **ASPM, BUB1, CASC5, CCNB2, CCNE2,** CDK2, **CDKN3,** CDKN1A, CDKN1B, C**ENPA, CKS2, COL5A2,** DSN1, ERBB2, **ESPL1,** HDAC1, IGFBP5, ITGB1, LGALS3, MIS12, **MKI67,** MYC, NDC80, NFYB, **POLE2,** PRSS3 (includes EG:5646), RB1, **SPC24,** TGFB1, **TOP2A,** TP53, TSPAN7, **WNT5B, ZWINT (includes EG:11130)** |
| Cell Morphology, Cell Death, Cell Cycle | 13 | 13 | **CCND2,** CLDN4, CTSC, DLEU1, **EFNB1**, FCGR1A, FCGR1B, FCGR1C, FST, **GCH1**, **GPR176**, HBEGF, **HJURP**, HSPB1, IgG, **INHBA**, KAT2B, KRT15, MRE11A, MYC, NBN, **NID1**, PMAIP1, PPP1R15A, **PRDM1**, **PTRF**, RND3, **SERPINB7**, SPIB, **TACC3**, TAPBP, TNF, TNFAIP3, **XRCC3** (**includes** **EG:7517**), **ZBTB16** |
| Cancer, Cellular Growth and Proliferation, Cell Cycle | 11 | 11 | **BRIP1**, CDC7, **CDH11**, CHEK1, **CLSPN**, CTNNB1, DBF4, DDX17, DKK1, DNMT3B, ESR1, **EXO1**, **EZH2**, FOSL2, **FOXM1**, KIF23, KLK3, **MCM2**, MLH1, NCOA3, NOS2, **PARD6B**, **PCSK6**, PDE4B, PPP3CA, RAD23A, RECQL, **RFC3**, SFN, SIRT1, TADA3, **TCF4**, TMEM97, TMSB15A (includes EG:11013), TP53 |
| DNA Replication, Recombination, and Repair, Cell Cycle, Cellular Assembly and Organization | 7 | 8 | AHSP, **ASF1B**, **AURKA**, AURKB, **CDC20**, CDKN2A, CHFR, **CHRNA5**, DIO1, **FBXO5**, FMR1, FZR1, G6PD, Histone h3, Histone h4, HNRNPK, HOXA9, KIR3DL1, LEP, MGMT, MTA2, **NCAPD2**, NCAPD3, NCAPG2, NCAPG (includes EG:64151), **NCAPH**, NCAPH2, NCL, PRLR, PTGS2, RASSF1, SMC2, **SMC4,** TGFBR1, THRAP3 |
| Antigen Presentation, Cell-To-Cell Signaling and Interaction, Cellular Growth and Proliferation | 1 | 1 | IFNB1, **SERPING1** |
| Carbohydrate Metabolism, Lipid Metabolism, Molecular Transport | 1 | 1 | **EEF1A2**, PI4KB |
| Cellular Growth and Proliferation, Cell Cycle, Cell Morphology | 1 | 1 | **CLCA2 (includes EG:9635),** ITGB4 |
| Antigen Presentation, Cell-To-Cell Signaling and Interaction, Hematological System Development and Function | 1 | 1 | ITGAM, **THY1** |
| Tissue Development, Dermatological Diseases and Conditions, Genetic Disorder | 1 | 1 | CSH1, ELN, **LOX** |
